# Doxorubicin and CpG loaded liposomal spherical nucleic acid for enhanced Cancer treatment

**DOI:** 10.1186/s12951-022-01353-5

**Published:** 2022-03-18

**Authors:** Bo Deng, Bing Ma, Yingying Ma, Pei Cao, Xigang Leng, Pengyu Huang, Yuanyuan Zhao, Tianjiao Ji, Xueguang Lu, Lanxia Liu

**Affiliations:** 1 Tianjin Key Laboratory of Biomedical Materials, Key Laboratory of Biomaterials and Nanotechnology for Cancer Immunotherapy, Institute of Biomedical Engineering, Chinese Academy of Medical Sciences & Peking Union Medical College, Tianjin, 300192 China; 2 CAS Key Laboratory for Biomedical Effects of Nanomaterials & Nanosafety, CAS Center for Excellence in Nanoscience, National Center for Nanoscience and Technology, Beijing, 100190 China; 3 Key Laboratory of Colloid, Interface and Chemical Thermodynamics, Institute of Chemistry, Chinese Academy of Science, No. 2, 1st North Street, Zhongguancun, Beijing, 100190 People’s Republic of China

**Keywords:** Nanoparticle, Co-delivery, Triggered release, CpG, Cancer immunotherapy

## Abstract

Chemotherapeutics that can trigger immunogenic cell death (ICD) and release tumor-specific antigens are effective on treating a variety of cancers. The codelivery of chemotherapeutics with adjuvants is a promising strategy to achieve synergistic therapeutic effect. However, low drug loading and complicated preparation of current delivery systems lead to carrier-associated toxicity and immunogenicity. Herein, we developed a facile approach to construct liposomal spherical nucleic acids (SNA) by the self-assembly of 1,2-dioleoyl-sn-glycero-3-phosphoethanolamine (DOPE)-doxorubicin conjugate and DOPE-matrix metalloproteinases-9 (MMP-9) responsive peptide-CpG conjugate (DOPE-MMP-CpG). Liposomal SNAs efficiently co-delivered DOX and CpG into tumors and released the two drugs upon biological stimuli of MMP-9 enzyme in tumor microenvironment (TME) and high concentration of endogenous glutathione in tumor cells. We demonstrated that liposomal SNA enhanced activation of dendritic cells (DCs), promoted expansion of CD8^+^ and CD4^+^ T cells in both tumors and spleen, inhibited tumor growth, and extended animal survival. This work provided a simple strategy of delivering chemotherapeutics and adjuvants to tumors with synergistic therapeutic effect and reduced side effect.

**Graphical Abstract:**

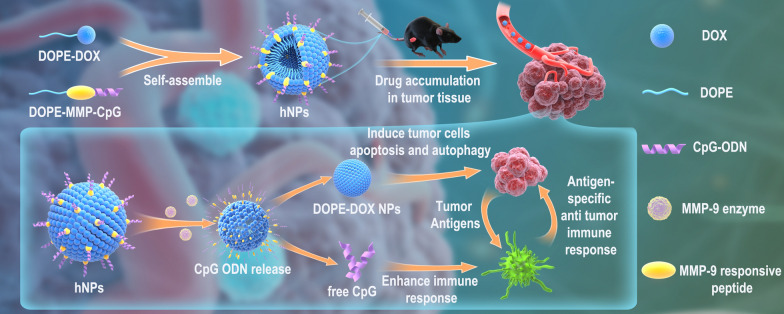

**Supplementary Information:**

The online version contains supplementary material available at 10.1186/s12951-022-01353-5.

## Introduction

Cancer vaccines, which harness the immune system to fight against cancer, have become one of the most promising therapies in clinic [1, 2]. Many cancer vaccines that are composed of tumor-associated antigens (TAAs) and adjuvants show promising efficacy on animal models and are currently under clinical investigation [3, 4]. Even though TAAs are highly expressed in tumors, they are still expressed in other healthy organs [5]. Therefore, TAA-specific T cells could attack normal tissue cells and cause severe side effects. Tumor specific antigen (TSA) is preferred to construct cancer vaccines [6]. However, it’s highly challenging to construct generic TSA because it varies significantly among patients [7]. Selected chemotherapeutics such as doxorubicin (DOX) could trigger immunogenic cell death (ICD) of cancer cells to release tumor-specific antigens [8, 9]. ICD-triggered immune responses could be amplified by immunostimulatory reagents, such as CpG oligodeoxynucleotides (CpG ODN) [10], which binds Toll-like receptor 9 (TLR-9) in the endosome and increases the infiltration of immune cells into TME [11, 12]. However, the delivery of CpG ODN into cells and TME is greatly hindered due to the inability of cell entry, poor stability, and rapid clearance of free CpG. A variety of studies utilized nanoparticles to co-deliver chemotherapeutics and adjuvants into tumors for synergistic therapy [13–16]. However, these nanoparticles are composed of mostly carrier materials such as inorganic nanoparticles or polymers [17, 18], resulting in low drug-loading efficacy, complex preparation, toxicity, and immunogenicity. These problems greatly hindered further translation into clinic [19]. Therefore, a simple system that can spatiotemporally deliver chemotherapeutics and adjuvants to tumors is still very much needed.

Spherical nucleic acids (SNAs) that consist of densely packed and highly oriented ODNs in a spherical geometry have emerged as efficient delivery vehicles for ODNs due to their great cellular uptake ability and enhanced stability against nuclease [20, 21]. CpG-functionalized SNA also showed superior TLR-9 activation compared to linear CpG, making SNA an attractive platform to construct vaccines. Currently, the development of SNA-based nanovaccine mainly focused on attaching TAA onto the surface of SNA for the codelivery of peptide or protein-based antigens and CpG adjuvant [22], therefore still facing the off-target immune response associated with TAAs. Additionally, the synthesis of liposome-based SNA utilizes a two-step approach, which involves liposome synthesis and then post-modification with cholesterol- or lipid-modified DNA. One-step synthesis of SNA by self-assembly of DNA amphiphile could simplify the preparation and enhance synthesis yield. For example, the use of SNA to co-deliver chemotherapeutics and antisense oligonucleotide was explored by conjugating DNA to polymeric DOX [23]. However, these studies aim to solve drug-resistance issue of cancer cells. The strategy of utilizing SNA to co-deliver chemotherapeutic and CpG to generate ICD and boost anticancer immune response has not been explored.

**Scheme 1:**
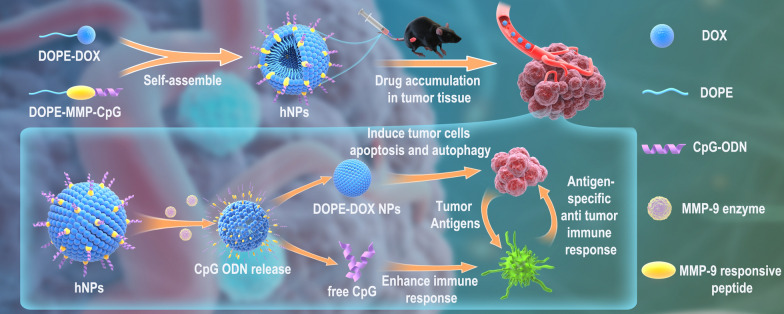
Schematic of DOX and CpG-loaded liposomal SNA and its mechanism of function

Herein, we developed a liposomal SNA by one-step self-assembly of lipid-DOX and lipid-CpG conjugates (Scheme 1). Disulfide bond and matrix metalloproteinases-9 (MMP-9)-responsive peptide were incorporated into lipid-DOX and lipid-CpG conjugates for bio-responsive release inside the cells and TME. We demonstrated that liposomal SNA could efficiently co-deliver and controlled release DOX and CpG in tumors and thus enhancing the direct killing effect of DOX on tumor cells as well as boosting potent tumor-specific immune responses to further eliminate tumor cells, achieving synergistic therapeutic effect with reduced systemic toxicity.

## Materials and methods

### Preparation and characterization of DOPE-DOX and DOPE-MMP-CpG

As shown in Additional file 1: Fig. S1, the conjugation of DOPE-MMP-CpG and DOPE-DOX was synthesized following our established protocols and some published literatures [24, 25]. DOPE-DOX was synthesized by conjugating DOX (Meilunbio, China) to 1,2-dioleoyl-sn-glycero-3-phosphoethanolamine (DOPE, Sigma-Aldrich Co, USA) through *N*-succinimidyl 3-(2-pyridyldithio) propionate (SPDP, Thermo Fisher Scientific Inc., USA) linker. Briefly, to synthesize DOPE-DOX, DOPE (6 mg) and DOX (4.7 mg) were separately reacted with *N*-succinimidyl 3-(2-pyridyldithio) propionate (SPDP, Thermo Fisher Scientific Inc., USA) (3 mg) at a molar ratio of 1:1.2 for 8 h at room temperature in 200 µl DMSO with catalytic amount of triethylamine (TEA), respectively. Then, the pyridyldithiol-activated DOPE was added with dl-dithiothreitol (DTT, Aladdin, China) (1.9 mg) in a 1.5:1 (DTT:DOPE) molar ratio and reacted for 3 h to be reduced into sulfhydryl-modified DOPE. The unreacted impurities of pyridyldithiol-activated DOX and sulfhydryl-modified DOPE were removed by 500 Da MWCO dialysis tubing. Finally, the resulted pyridyldithiol-activated DOX and sulfhydryl-modified DOPE were mixed and stirred for 24 h at room temperature. The unreacted impurities were removed by 1 kDa MWCO dialysis tubing.

The long molecule DOPE-MMP-CpG was synthesized with adjuvant molecules CpG-ODN (Type C 2395, sequence: 5′-TCGTCGTTTTCGGCGCGCGCCG-3′, Sangon Biotech, China), matrix metalloproteinase-9 (MMP-9) responsive peptides (MMP, sequence: GPQGIAGQR, ChinaPeptides Co., Ltd, Shanghai) and DOPE. The synthesis of DOPE-MMP-CpG involved four steps. First, MMP (0.26 mg) were pyridyldithiol-activated by SPDP (0.11 mg) at a molar ratio of 1:1.2 for 8 h at room temperature in 300 µl DMSO under the presence of TEA. The unreacted impurities were removed by 500 Da MWCO dialysis tubing. Then, the resulted pyridyldithiol-activated MMP were reacted with an equal amount sulfhydryl-modified CpG-ODN (50 OD) for 24 h at room temperature and conjugated by disulfide bonds. The unreacted impurities were removed by 7 kDa MWCO dialysis tubing. Next, the carboxyl groups of MMP-9 responsive peptides were activated by a suitable amount 1,2-bichloroethane (EDC, Sigma-Aldrich Co., USA) (0.66 mg) and *N*-hydro-xysuccinimide (NHS) (0.3 mg) for 4 h at 40 ℃ in 200 µl DMSO. The unreacted impurities were removed by 7 kDa MWCO dialysis tubing. At last, the activated carboxyl groups of MMP-9 responsive peptides and the amino groups of DOPE were allowed to react at a molar ratio of 1:1.2 at room temperature for 24 h in 300 µl DMSO. DOPE-MMP-CpG were synthesized through the amide-forming reaction. The unreacted impurities were removed by dialysis using 7 kDa MWCO dialysis tubing.

The synthesis of DOPE-MMP-CpG was verified by agarose gel electrophoresis and further determined using Fourier Transform Infrared Spectrometer (FTIS). The MMP-9 enzyme responsiveness of DOPE-MMP-CpG was evaluated by agarose gel electrophoresis. DOPE-DOX were characterized by FTIS.

### Preparation and characterization of hNPs

The DOPE-DOX or DOPE-MMP-CpG with amphiphilic properties was self-assembled itself (named as DOPE-DOX NPs and DMC NPs) or the two molecules self-assembled at ratios of 10:1, 20:1, 30:1 (named as hNPs), respectively. To prepare the hNPs, the lyophilized powder of DOPE-DOX NPs and DMC NPs were precisely weighed with a certain proportion. The lyophilized powder was dissolved in a small amount of DMSO and the solutions were diluted with water to a final concentration of 1% DMSO. The solution was sonicated for 30 s and then stirred at 25 °C for 2 h to obtain hNPs by self-assembly. The size and zeta potential of nanoparticles were detected by dynamic light scattering (DLS). The surface morphology was observed by transmission electron microscope (TEM, JEOL JEM-100CX-II, Japan). The encapsulation efficiencies of DOX and CpG were evaluated after hNPs were centrifuged at 18 000 × rpm for 20 min.

To evaluate the responsiveness of hNPs to TME, hNPs were digested by MMP-9 enzyme (2 µg/ml) for 5 min and observe their size alteration by DLS. To detect the DOX release behavior from hNPs, nanoparticles were dissolved in PBS with DTT and MMP-9 enzyme in a 1 kDa dialysis tubing and measure the concentration of DOX in dialysate at preset timepoints at 37 ℃.

### In vitro experiments

#### Cytotoxicity assessment

Human umbilical vein endothelial cells (HUVEC) were cultured with endothelial cell medium (93% basal medium + 5% fetal bovine serum (FBS) + 1% endothelial cell growth supplement + 1% penicillin/streptomycin solution) under 5% CO_2_ at 37 ℃. To evaluate the cytotoxicity of hNPs to HUVEC, HUVEC were seeded into 96-well culture at a density of 10^4^ cells/well and co-incubated with hNPs and free DOX with serious dilutions for 24 h. The cytotoxicity of hNPs was determined by CCK-8 kit (CCK-8, Dojindo Molecular Technologies, Inc., Japan) according to manufacturer’s protocol.

#### Cellular uptake of DOX

Tumor cell lines E.G7-OVA were co-incubated with hNPs or free DOX (DOX concentration was 2 µM) for 2 h, 4 h and 6 h, respectively. Cells were washed with PBS three times before staining with DAPI and fixation for 20 min. The cells were then imaged by Confocal Laser Scanning Microscopy (CLSM, Zeiss LSM 800, Germany). Fluorescent images were quantified by ImageJ.

#### BMDC activation and maturation

Tumor cells E.G7-OVA were treated with PBS, free DOX, free DOX and CpG and hNPs (DOX concentration was 2 µM, CpG-ODN concentration was 10 µg/ml) for 24 h, respectively. Then the treated dying cell or debris were collected and used for the following experiment. Bone Marrow-Derived Dendritic Cells (BMDCs) collected from femur of C57BL/6 mice and induced with GM-CSF (20 ng/ml) and IL-4 (10 ng/ml) under 5% CO_2_ at 37 ℃. After a week of cultivation, BMDCs were co-cultured with the above various treated E.G7-OVA debris for 48 h. After stained with cy5.5-labeled CD11c, FITC-labeled CD86, APC-labeled MHC and PE-labeled CD40 antibodies (eBioscience, CA, USA) for 30 min. The expression of MHC molecules and co-stimulating molecules on BMDCs were evaluated with a flow cytometer. In the meantime, cytokines (IL-1β, IFN-γ and TNF-α) in the culture supernatant were assessed by ELISA kits (Thermo Fisher Scientific Inc., USA).

### In vivo immunization experiments

#### Biodistribution experiment

The animal use protocol has been reviewed and approved by the Animal Ethical and Welfare Committee (AEWC, Approval No. IRM-DWLL-2021074) of Institute of Radiation Medicine Chinese Academy of Medical Sciences & Peking Union Medical College. In order to detect the biodistribution of hNPs after intravenous injection in mice, DOPE was conjugated with fluorescence cy7 instead of DOX to prepare nanoparticles named as cy7-hNPs. Cy7-hNPs nanoparticles and free Cy7 were injected intravenously (20 µg/mouse) into the C57BL/6 mice (Beijing WTLH Laboratory Animal Technology Co., Ltd, Beijing, China), respectively. The in-vivo Maestro imaging system (IVIS, Maestro EX, USA) was used to monitor the fluorescence signal at various time points in mice. At 48 h after inject, the mice were sacrificed and tumors and organs were collected for fluorescence imaging and quantified the fluorescence intensity by CRI.

#### Therapeutic effect

To establish tumor xenograft models, 5 × 10^5^ E.G7-OVA cells were implanted subcutaneously into the right back of 6-week-old female C57BL/6 mice. Tumor*-*bearing E.G7-OVA mice were randomized into 4 groups until the volume of tumor reached nearly 50 mm^3^. Then, mice were injected with PBS, free DOX, free DOX and CpG and hNPs (n = 6, DOX concentration was 0.1 mg/mouse, CpG-ODN concentration was 40 µg/mouse), respectively, and the treatments were performed 3 times at intervals of 6 days.

To assess the therapeutic efficacy, the body weight of mice, tumor volumes and survival period were recorded every day. Mice were deemed as death when the tumors volume was larger than 2000 mm^3^ volume.

In order to further evaluate the immune effect of hNPs and explore the related mechanism, mice were sacrificed at 72 h after the third therapy, and their spleens, hearts, lymph nodes and tumors were harvested.

#### Histopathological evaluation of tumor and myocardium

To evaluate the tumor apoptosis and the cardiovascular toxicity of hNPs. The tumor and heart tissue sections of mice were stained with H&E (hematoxylin and eosin) for histopathological evaluation.

#### T cell immune responses

Lymphocytes were isolated from lymph nodes and spleens by lymphocyte separation solution and were processed into single-cell suspension. The lymphocytes were co-cultured with fluorescence-labeled antibodies against CD8, CD4 and CD3 to assess the magnitude of immune response.

#### Cytokine secretion

2 × 10^5^ splenic lymphocytes from various treated mice were seeded into 96-well plates and re-stimulated with dying tumor cells or debris for 48 h at 37 ℃. After centrifugation at 450 g for 5 min, the supernatants were harvested to assess the cytokine expression levels of IL-1β, IL-18, IFN-γ and TNF-α with ELISA kit.

#### Immune memory

Splenic lymphocytes from mice treated with various formulations were re-stimulated with dying tumor cell or debris for 72 h and stained using different fluorescent CD62L, CD44, CD4 and CD8 antibodies (eBioscience, CA, USA). The proliferation of memory T cells were detected with flow cytometry.

### Statistical analysis

All data were presented as mean result ± standard deviation (SD). Statistical significance of differences was analyzed using Student’s t-test or ANOVA analysis. P-value less than 0.05 was considered as statistically significant.

## Results and discussion

### hNPs preparation and characterization

**Fig. 1:**
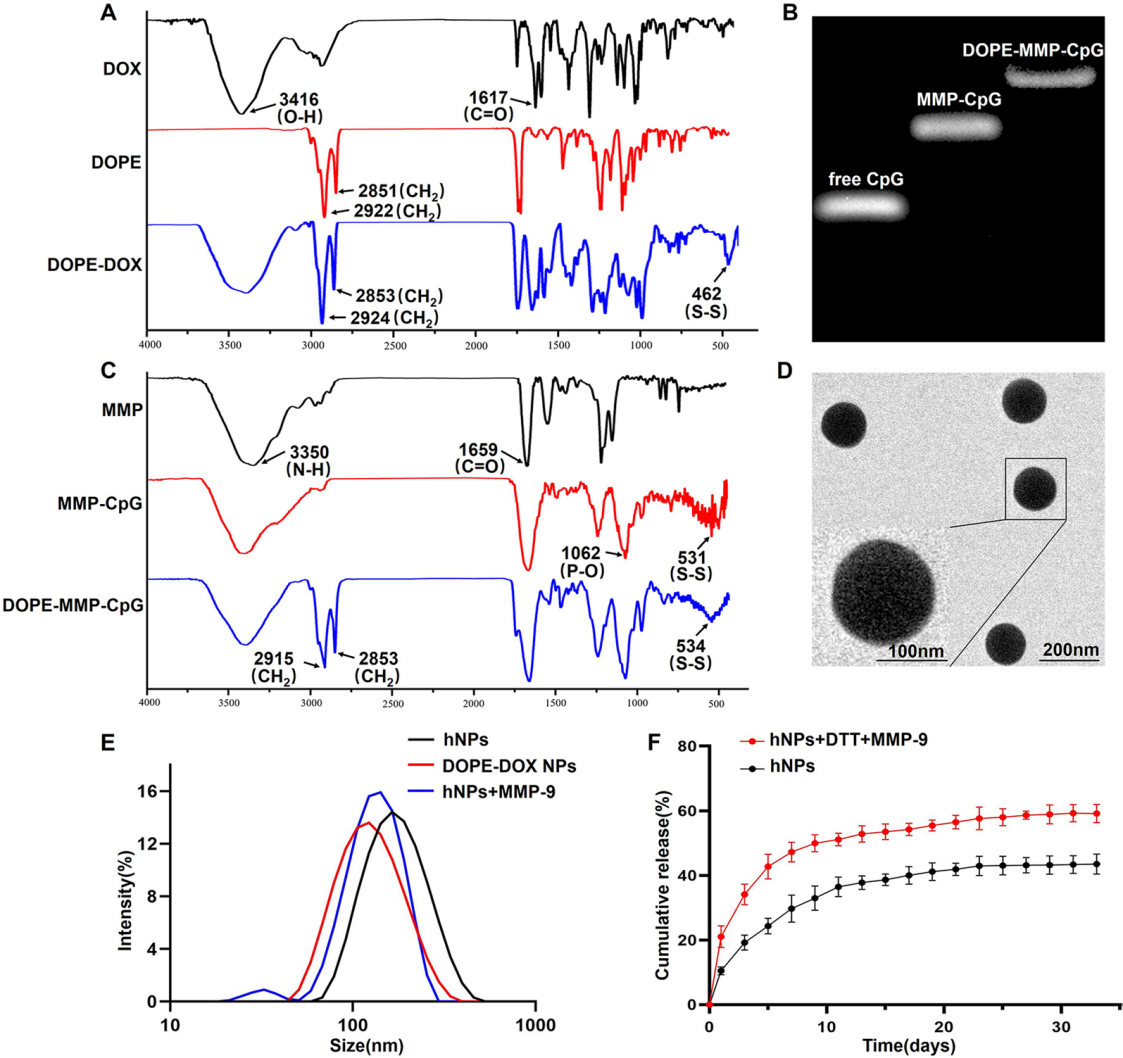
**A** FTIR spectra of DOX, DOPE, and DOPE-DOX. **B** Agarose gel electrophoresis image and **C** FT-IR spectra of MMP, MMP-CpG, and DOPE-MMP-CpG. **D** Representative TEM images of hNPs. **E** DLS measurements of hNPs before and after incubation with MMP-9 enzyme. DOPE-DOX NPs were used as a control. **F** DOX release profile from hNPs in PBS without/with MMP-9 enzyme and DTT

To achieve co-deliver and bio-responsive release of DOX and CpG, we prepared liposomal-SNA by self-assembly of lipid-DOX and lipid-CpG conjugates. FDA-approved DOPE was selected as the only carrier material. To synthesize DOPE-DOX conjugate, DOPE and DOX were separately reacted with SPDP to convert their amino groups to pyridine disulfide groups. DOPE-pyridine disulfide was treated with DTT to yield thiol-modified DOPE, which was then reacted with DOX-pyridine disulfide to yield DOPE-S-S-DOX conjugate. The final conjugate was dialyzed against water to remove unreacted DOX. DOPE-S-S-DOX was characterized by Fourier-transform infrared spectroscopy (FT-IR). The absorption peaks of DOPE-S-S-DOX at 2852 cm^−1^ and 2923 cm^−1^ were designated to the CH_2_ stretching vibration, which was not observed in the spectrum of free DOX. A new absorption peak emerged at 462 cm^−1^, which was the characteristic absorption peak of disulfide bond (Fig. 1A). These results demonstrated the successful synthesis of DOPE-S-S-DOX.

DOPE-CpG conjugate was synthesized through an MMP-9-responsive peptide (MMP, sequence: GPQGIAGQR). The N-terminal of peptide was activated by SPDP to yield pyridine disulfide-modified peptide, which was then allowed to react with thiol-modified CpG through disulfide exchange reaction to yield MMP-CpG conjugate. The carboxylic group at the C-terminal of peptide was reacted with EDC and NHS to yield NHS-modified MMP-CpG conjugate. Lastly, DOPE was coupled to the C-terminal of peptide through amidation reaction. Successful synthesis of DOPE-MMP-CpG was proved by FT-IR and agarose gel electrophoresis. There were new absorption peaks at 531 cm^−1^ and 534 cm^−1^ of MMP-CpG and DOPE-MMP-CpG, respectively, which were attributed to the disulfide bond of CpG-MMP. Additionally, DOPE contained a large amount of methylene. New peaks for methylene (CH_2_) at 2915 cm^−1^ and 2853 cm^−1^ in DOPE-MMP-CpG proved that DOPE was covalently connected to MMP-CpG (Fig. 1C). Agarose gel electrophoresis showed that the molecular sizes of CpG, MMP-CpG and DOPE-MMP-CpG gradually increased, suggesting successful reaction of each step (Fig. 1B). Additionally, there was no free CpG or MMP-CpG in the final DOPE-MMP-CpG conjugate. The MMP-9 enzyme responsiveness of DOPE-MMP-CpG was confirmed by agarose gel electrophoresis (Additional file 1: Fig. S2).

We next studied the co-assembly of DOPE-DOX and DOPE-MMP-CpG to yield nanoparticles. Different ratios of DOPE-MMP-CpG and DOPE-DOX were allowed to self-assemble in water to form nanoparticles. The hydrodynamic diameter and size distribution of nanoparticles were measured by DLS. As shown in Additional file 1: Fig. S3, DOPE-DOX or DOPE-MMP-CpG could self-assemble into nanoparticles by themselves. They could also form stable hybrid nanoparticles (hNPs) at different ratios. According to the dose of DOX and CpG ODN in the following in vivo experiments, the hNPs at the ratio of 30:1 (DOPE-DOX: DOPE-MMP-CpG) was used for following experiments. The diameter of hNPs was ~ 160.6 ± 1.3 nm (PDI = 0.126 ± 0.05) and the zeta potential was − 25.3 ± 0.9 mV. TEM images showed that hNPs exhibited spherical morphology with uniform size distribution (Fig. 1D). The encapsulation efficiencies for DOX and CpG were ~ 92% and ~ 84%, respectively. As shown in Additional file 1: Figs. S4 and S5, hNPs remained stable for 3 weeks in PBS and 1 week in RPMI medium containing 10% FBS, respectively. These results demonstrated that DOPE-DOX and DOPE-MMP-CpG could form stable hybrid nanoparticles.

To evaluate the MMP-9 enzyme responsiveness of hNPs, the size alteration of hNPs was measured by DLS before and after incubation with MMP-9 enzyme. The diameter of hNPs became clearly smaller after incubation with MMP-9 and was similar to that of DOPE-DOX NPs (Fig. 1E), indicating that hNPs released CpG ODN upon peptide cleavage. The remaining DOPE-DOX maintained stable as nanoparticles. These results suggested that hNPs could be cleaved in TME with a high level of MMP-9 enzyme and release CpG ODN to stimulate antigen presenting cells.

The release of DOX from hNPs was evaluated in PBS containing 0.01 M DTT to mimic the reducing intracellular environment of tumor cells. The results showed that ~ 34.1% of DOX burst released in the first 3 days, and then constantly and slowly released up to 59.9 ± 2.5% over 31 days (Fig. 1F). The sustained DOX-release profile suggested that DOPE-DOX NPs could kill tumor cells for a long period of time.

### In vitro experiments

#### Cytotoxicity assessment

**Fig. 2:**
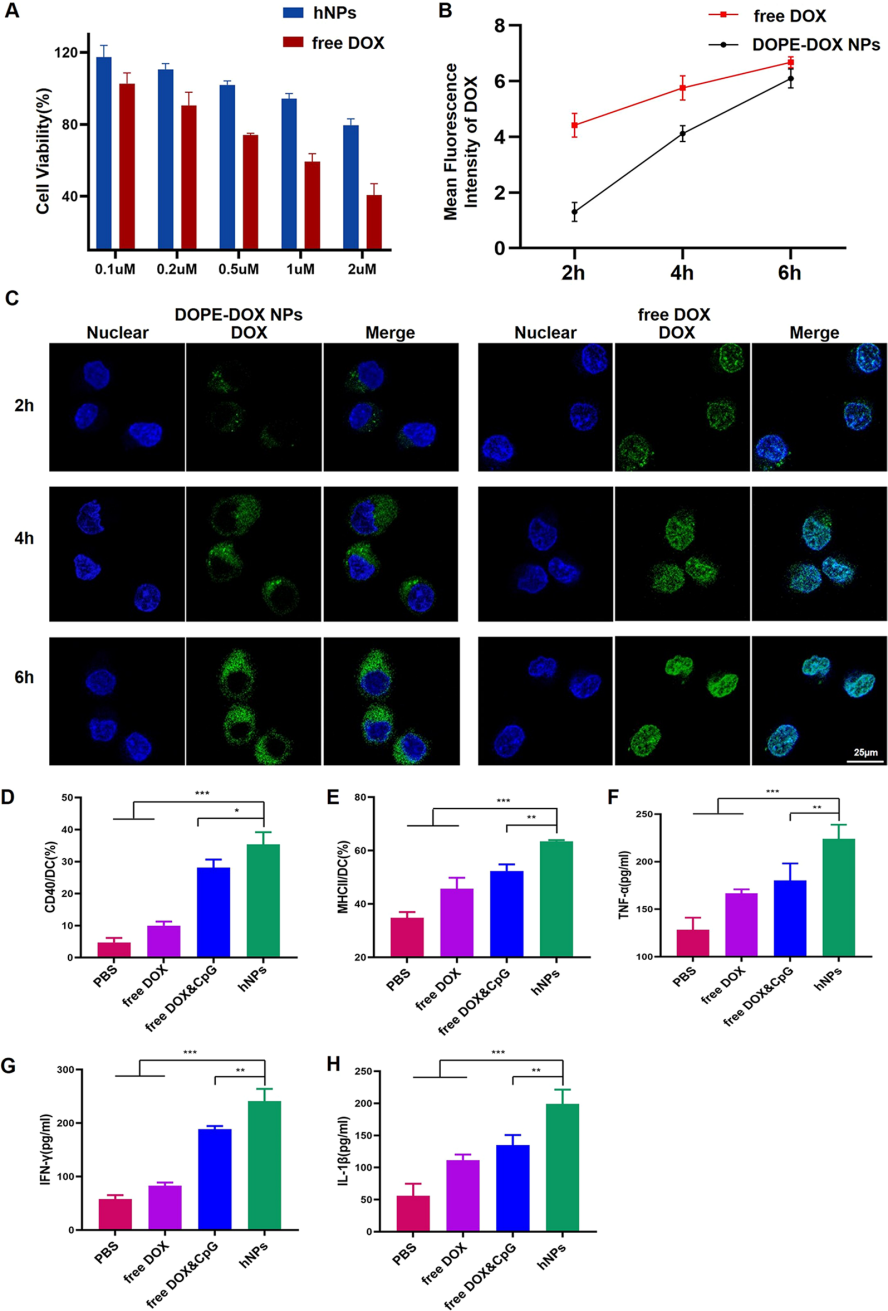
**A** Cell viability of HUVEC after incubation with various concentrations of hNPs or free DOX and CpG. **B** Quantification of the cellular uptake of DOX by E.G7-OVA cells. **C** Representative CLSM images of E.G7-OVA cells after incubation with DOPE-DOX NPs or free DOX for 2 h, 4 h and 6 h, respectively. The molecule expression of **D** CD40 and **E** MHC-II was analyzed by flow cytometry. The cytokine expression of **F** TNF-α, **G** IFN-γ and **H** IL-1β were analyzed by ELISA assays. Results represent mean ± SD (n = 6; *P < 0.05, **P < 0.01, ***P < 0.001)

We first assessed whether hNPs could induce less cytotoxicity against normal endothelium cells than free DOX. HUVEC were treated with hNPs at different concentrations for 24 h. Cell viability was evaluated by CCK-8 assay. Free DOX decreased cell viability to ~ 40% at the concentration of 2 µM. On the other hand, cell treated with hNPs showed significant higher cell viability than free DOX at all tested concentrations (Fig. 2A), indicating that hNPs possessed better safety profile than free DOX.

#### Uptake of DOX

When hNPs reached tumor site where MMP-9 enzyme is highly expressed, these nanoparticles would release CpG ODN to stimulate immune responses. The remained DOPE-DOX NPs is expected to kill tumor cells. Several studies demonstrated that free DOX and DOX-loaded nanoparticles differed in their subcellular distribution and the way they caused tumor cell death [26, 27]. Free DOX intercalated into DNA in nuclei resulting in cell oxidative damage and induced ICD-triggered immune responses. DOX-loaded nanoparticles primarily stayed in the cytoplasm and induced mutual reinforced loop between autophagy and released high-mobility group box protein B1 (HMGB1), which can elicit powerful immune responses. We studied the cellular uptake of DOPE-DOX NPs in a lymphoma tumor cell line E.G7-OVA. Free DOX or DOPE-DOX NPs were incubated with E.G7-OVA cells for different periods of time before imaging by CLSM. The cellular uptake amounts of free DOX and DOPE-DOX NPs were similar at 6 h post incubation (Fig. 2B). However, the intracellular distribution of DOX is highly different. Most of DOPE-DOX NPs appeared in cytoplasm. Most of free DOX located in nuclei (Fig. 2C). The results of intracellular DOX distribution combined with the profile of DOX-release indicated that DOPE-DOX NPs could induce both ICD and autophagy-triggered immune responses.

#### BMDC activation and maturation

The maturation and activation of dendric cells (DCs) is the key to initiate strong immune responses. We evaluated whether tumor cells treated with hNPs could induce DC maturation and activation. To mimic the process in TME, BMDCs from mice were incubated for 48 h with E.G7-OVA cells, which were pre-treated with hNPs (treated with MMP-9 enzyme beforehand), free DOX and CpG, or free DOX for 24 h, respectively. The concentrations of DOX and CpG in each group were 2 µM and 10 µg/ml, respectively. E.G7-OVA cells treated with PBS were utilized as the negative control. BMDCs were labeled with antibodies against CD11C, MHC-II, CD86 and CD40 and evaluated by flow cytometry. Cytokine (IL-1β, IFN-γ and TNF-α) levels in the culture supernatant were assessed by ELISA kits. The results showed that expression of MHC-II and co-stimulatory molecules CD40 in the hNPs group were dramatically enhanced compared with free DOX and CpG and free DOX (Fig. 2D, E and Additional file 1: Fig. S6), indicating that hNPs could promote BMDCs maturation. The secretion of IFN-γ and IL-1β by hNPs-treated DCs was significantly enhanced compared with other groups (Fig. 2G, H). The secretion of TNF-α, which plays a crucial role in the proliferation of T cells, was elevated over 200% by hNPs treatment in comparison to PBS group (Fig. 2F), indicating hNPs remarkably elicited and amplified the immune responses. These results suggested that hNPs could effectively induce ICD of tumor cells and facilitate production of IFN-γ to trigger T cell immune responses. Collectively, these results demonstrated that hNPs could facilitate DCs activation and maturation to invoke tumor-specific immune responses.

### In vivo immunization experiments

#### Biodistribution of hNPs

**Fig. 3:**
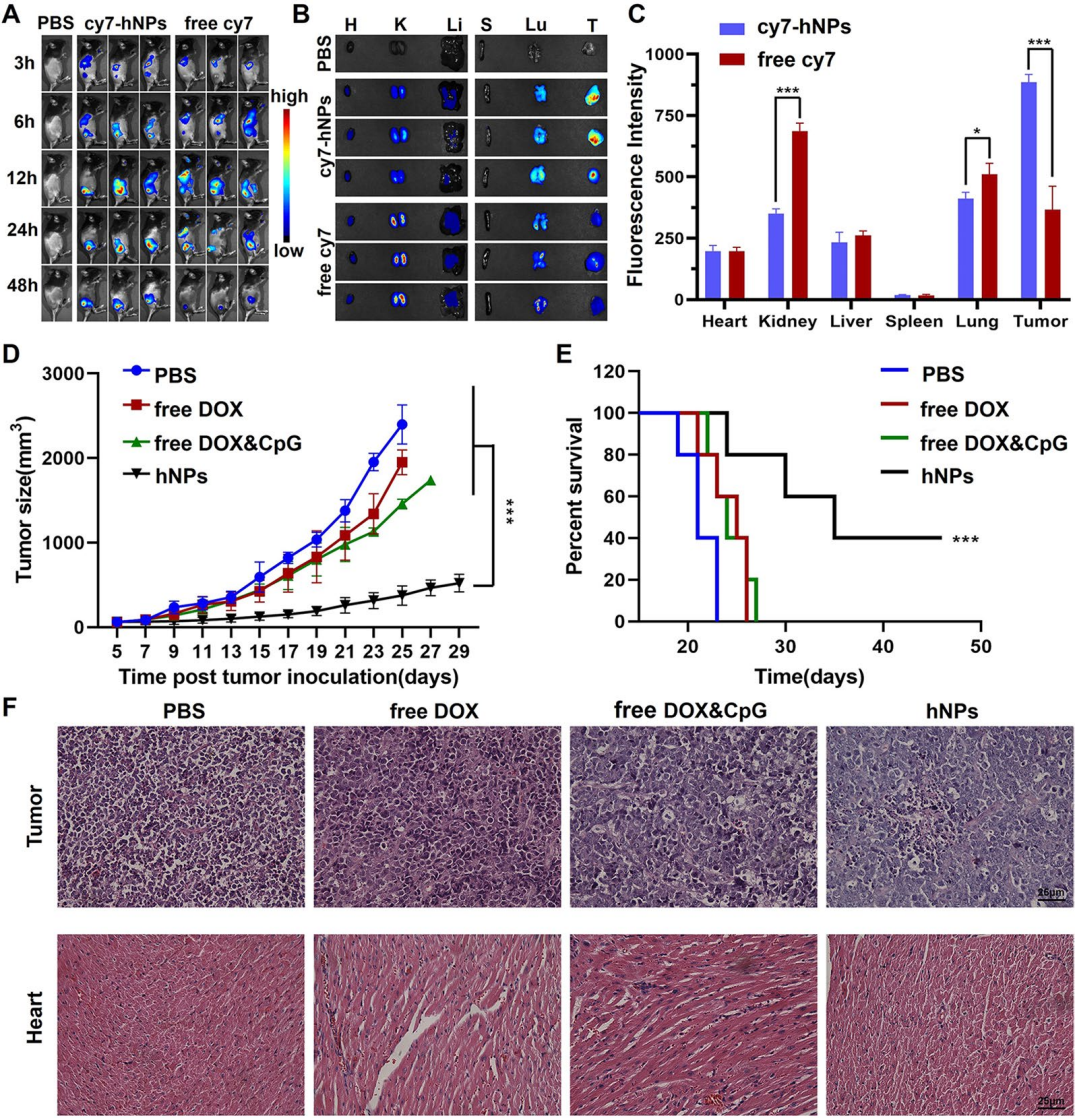
The biodistribution and therapeutic effect of hNPs in E.G7-OVA tumor-bearing mice. **A** IVIS images of mice at selected time-points after intravenous injections of cy7-hNPs or free Cy7. **B** The fluorescence images of organs and tumors collected from mice at 48 h post injection and **C** quantified mean fluorescence intensity. Tumor growth (**D**) and animal survival curves (**E**) of mice treated with PBS, free DOX, free DOX and CpG or hNPs, respectively. **F** H&E staining images of tumor and heart from mice treated with various formulations. Data represent mean ± SD (n = 5; *P < 0.05, ***P < 0.001)

Systemic administration of free DOX not only causes off-target toxic effects, but also reduces the local concentration of drugs at the tumor site accompanied by weak induction of ICD. Nanoparticles are expected to offer superior accumulation in local tumor tissues and less adverse side effects than conventional chemotherapeutic drugs [28–30]. To determine whether these hNPs could promote drug accumulation at tumor site after intravenous injection, fluorescent Cy7 instead of DOX was used to construct hybrid nanoparticles (cy7-hNPs). Cy7-hNPs or free Cy7 was administered into tumor bearing mice by intravenous injection. The biodistribution of Cy7-hNPs in tumor-bearing mice was imaged using the Maestro imaging system. Free Cy7 continually accumulated in the liver from 3 to 12 h. A very small amount of free Cy7 accumulated in tumor at 6 h. Free Cy7 gradually increased until 24 h, and then decreased at 48 h post injection. In contrary, Cy7-hNPs showed much higher accumulation at tumor site at 6 h than free Cy7 after injection and the fluorescent intensity kept constantly high at 24 and 48 h compared to free Cy7 (Fig. 3A). Several studies have shown that nanoparticles can cross the blood brain barrier and affect nervous system [31–33]. However, we did not observe any fluorescent signals in the brain. This is reasonable because most nucleic acid-based nanoparticles or spherical nucleic acids do not have the ability to cross blood brain barrier without targeting ligands. Further design and modification are needed to use hNPs for brain delivery. These results indicated that hNPs could decrease the absorption of Cy7 by liver and improve tumor accumulation.

At 48 h post intravenous injection, tumors and organs were collected for ex vivo fluorescence imaging to quantify the biodistribution of Cy7-hNPs. As shown in Fig. 3B, C, free Cy7 accumulated the most in the kidney but smaller amounts in the lung, liver, and tumor, suggesting rapid renal clearance. Cy7-hNPs showed ~ 2.4-fold increase of tumor accumulation. Such observation is consistent with previous studies that showed small molecules or nanostructures were mainly cleared by reticuloendothelial system [34], while nanoparticles with diameter ~ 30 nm showed efficient tumor passive accumulation [35]. Even though hNPs still accumulated in major organs such as lung and liver, the majority of hNPs accumulated in tumors [36]. The current hNPs did not have significant selectivity to tumors than other major organs. Previous studies showed that small molecules or nanoparticles with diameter smaller than ~ 6 nm were mainly cleared through kidney [37, 38]. The improved tumor accumulation of hNPs than free dye was probably due to the increased size, which diminished renal clearance and leaved more drugs available in blood circulation. The targeting ability to tumor could be achieved by conjugating antibodies or aptamers that can selectively bind to tumor [39–42]. Collectively, these results demonstrated that hNPs greatly increased drug accumulation in the tumor.

#### The therapeutic effect of hNPs

To evaluate the in vivo tumor inhibition effect of hNPs, an E.G7-OVA tumor model was established and treated with three intravenous administrations of hNPs, PBS, free DOX, or free CpG and DOX, respectively. As shown in Fig. 3D, E, hNPs group showed the most potent tumor inhibition effect compared with other groups. Furthermore, the average survival of mice treated with PBS, free DOX and free DOX and CpG were 21.4 d, 24.2 d, and 24.4 d, respectively. hNPs significantly exceeded survival time to 36.6 d. 80% of mice treated with hNPs survived over 30 days and 40% survived over 45 days. Moreover, hNPs were less toxic than free DOX or DOX and CpG. Mice treated with free DOX or free DOX and CpG showed significant weight loss (Additional file 1: Fig. S7). The body weight of mice treated with hNPs remained constant. The results of H&E staining further confirmed that hNPs induced clear apoptosis in tumor tissue with reduced cardiovascular toxicity compared to free DOX and free DOX and CpG (Fig. 3F). These results demonstrated that hNPs exhibited prominent anti-tumor effect with good safety profile.

#### Analysis of T cells activation

**Fig. 4:**
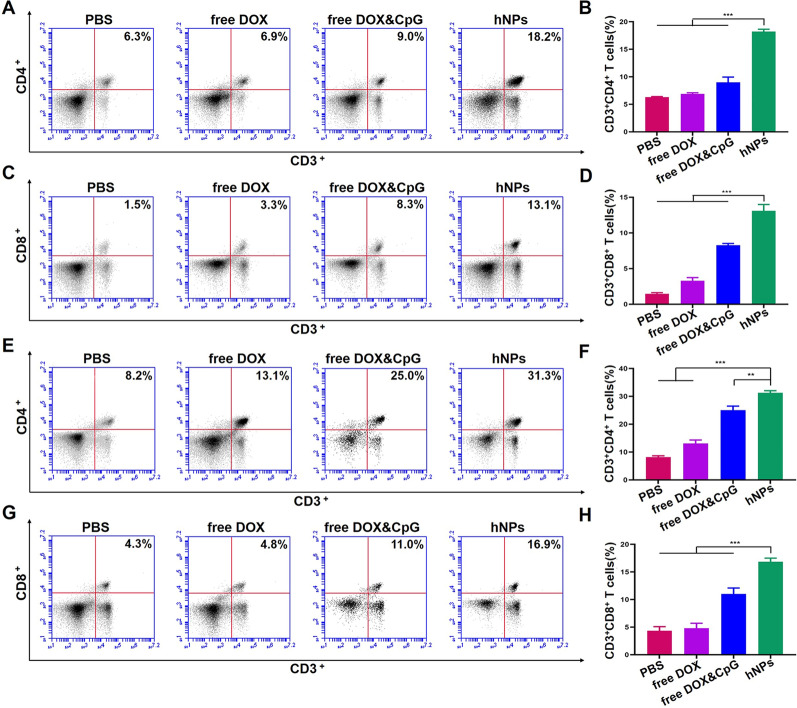
hNPs effectively enhanced proliferation of CD3^+^CD4^+^ and CD3^+^CD8^+^ T cells. **A** Representative FACS plots and **B** percentages of CD3^+^CD4^+^ and CD3^+^CD8^+^ (**C**, **D**) T cells in lymph nodes. **E** Representative FACS plots and **F** percentages of CD3^+^CD4^+^ and CD3^+^CD8^+^ (**G**, **H**) T cells in spleen lymphocytes. Data represent mean ± SD (n = 6; **P < 0.01, ***P < 0.001)

To evaluate the activation of immune responses of hNPs in vivo, we assessed the proliferation of lymphocytes in lymph nodes and spleen after treatments with hNPs, free DOX, free DOX and CpG, and PBS, respectively. Compared to PBS, the percentage of CD3^+^CD4^+^ T cells in lymph nodes treated with hNPs enhanced ~ 3 times (Fig. 4A, B). The percentage of CD3^+^CD8^+^ significantly enhanced ~ 9 times (Fig. 4C, D). The lymphocytes in spleen also showed significant enhanced proliferation (Fig. 4E–H). The percentages of CD3^+^CD4^+^ T cells and CD3^+^CD8^+^ T cells reached 31.3% and 16.9% in hNPs group, respectively, which were ~ 4-fold compared to PBS group. These results indicated that hNPs promoted proliferation of CD4^+^CD3^+^ and CD8^+^CD3^+^ T cells in lymph nodes and spleen.

#### Mechanism analysis

**Fig. 5:**
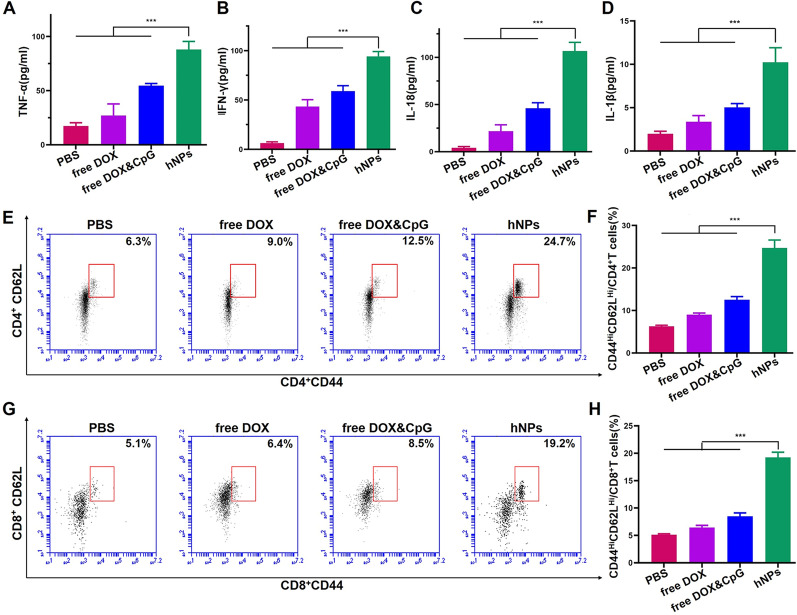
The cytokine expression of **A** TNF-α, **B** IFN-γ, **C** IL-18 and **D** IL-1β in supernatant of spleen lymphocytes from various treated mice, which were re-stimulated with E.G7-OVA fragments. **E**–**H** Expression of CD62L and CD44 on CD4^+^ or CD8^+^ T cells were evaluated using Flow cytometry. Data represent mean ± SD (n = 6; ***P < 0.001)

Previous studies showed that free DOX could serve as an apoptosis inducer and initiate ICD triggered immune responses through inflammasome pathway. DOX-loaded nanoparticle induced autophagy and released HMGB1, which could promote Th1-type immune responses [43]. In addition, CpG amplifies innate and adaptive T-cell immune responses by releasing inflammatory cytokines including TNF-α and IFN-γ, which could further enhance the anti-tumor immunity. To verify how hNPs enhance DOX-triggered immune responses and explore the underlying mechanism, the lymphocytes in spleens were collected after various treatments and restimulated with E.G7-OVA fragments for 72 h. The expression level of inflammasome pathway-related cytokines (IL-1β and IL-18) and Th1-type cytokines (TNF-α and IFN-γ) in cell culture supernatant were detected. As shown in Fig. 5A–D, hNPs remarkably improved the level of IL-1β and IL-18 and increased the secretion of TNF-α and IFN-γ compared to free DOX and free DOX and CpG treated groups. The levels of TNF-α and IFN-γ were enhanced by ~ 5 and ~ 14 times compared to PBS group, respectively. Collectively, these results suggested that hNPs significantly enhanced the apoptosis and autophagy of tumor cells and induced strong antitumor immune responses through both ICD-induced and Th1-type immune responses.

#### Memory immunity

Encouraged by the potent systemic immune response of hNPs in lymph nodes and spleen, we evaluated the induction of central memory T cell (T_cm_, CD62L^hi^CD44^+^ T cell) [44]. T_cm_ could persist for a long duration and have rapid recall ability to recognize old-antigens and prime antigen-specific immune responses to prevent tumor recurrence [45]. Lymphocytes from the spleen of immunized mice were re-stimulated with E.G7-OVA fragments. The cells were stained with CD62L and CD44 antibodies and detected with flow cytometry. The results demonstrated that hNPs induced remarkable expansions of both CD4^+^ T_cm_ and CD8^+^ T_cm_. The percentage of CD4^+^ T_cm_ in hNPs group was enhanced to 24.7% compared to PBS group (6.3%), free DOX (9.0%), and free DOX and CpG (12.5%) (Fig. 5E, F). hNPs also remarkedly elevated the proliferation of CD8^+^ T_cm_ compared to other groups (Fig. 5G, H). These results indicated that hNPs could induce anti-tumor memory immunity, therefore holding a potential of inhibiting tumor recurrence.

## Conclusions

In summary, we developed a facile strategy to construct liposome-based SNA through one-step, co-assembly of lipid-drug and lipid-DNA conjugates. Such hybrid nanoparticle co-delivered and released DOX and CpG upon biological stimuli in tumors. hNPs activated both ICD-induced immune responses and autophagy mediated Th1-type immune responses, increased DC activation efficacy, increased CD8^+^ and CD4^+^ T cell population in tumors, effectively inhibited tumor growth, and extended animal survival. Additionally, this nanoparticle reduced the systemic toxicity of DOX and employed FDA-approved DOPE as the only carrier material, therefore could serve as an effective and safe cancer therapy. Overall, this work provided a simple design strategy of delivering chemotherapeutics and adjuvants for cancer immunotherapy.

## Supplementary Information

**Additional file 1: Figure S1.** The chemical synthesis scheme of DOPE-DOX and DOPE-MMP-CpG. **Figure S2.** The agarose gel electrophoresis image of free CpG, DOPE-MMP-CpG & MMP9 enzyme and DOPE-MMP-CpG. **Figure S3.** Distribution of particle size at various proportions in water. **Figure S4.** The stability assessment of hNPs in PBS. **Figure S5.** The stability assessment of hNPs in RPMI medium containing 10% FBS. **Figure S6.** After co-incubated with dying tumor cells or debris treated with various formulations for 48 h, the expression level of CD86 on BMDC was analyzed by flow cytometry. **Figure S7.** Body weight change of mice treated with PBS, free DOX, free DOX and CpG and hNPs, respectively.

## Data Availability

All data are included in this published article.
