# Supplementary material for: Doxorubicin and CpG loaded liposomal spherical nucleic acid for enhanced Cancer treatment
Source: J Nanobiotechnology. 2022 Mar 18;20:140. doi: 10.1186/s12951-022-01353-5 (PMC8932194; doi:10.1186/s12951-022-01353-5)
Supplement: Supplementary file 1 — Additional file 1: Figure S1. The chemical synthesis scheme of DOPE-DOX and DOPE-MMP-CpG. Figure S2. The agarose gel electrophoresis image of free CpG, DOPE-MMP-CpG & MMP9 enzyme and DOPE-MMP-CpG. Figure S3. Distribution of particle size at various proportions in water. Figure S4. The stability assessment of hNPs in PBS. Figure S5. The stability assessment of hNPs in RPMI medium containing 10% FBS. Figure S6. After co-incubated with dying tumor cells or debris treated with various formulations for 48 h, the expression level of CD86 on BMDC was analyzed by flow cytometry. Figure S7. Body weight change of mice treated with PBS, free DOX, free DOX and CpG and hNPs, respectively. [file 12951_2022_1353_MOESM1_ESM.docx]

Additional file 1

**Doxorubicin and CpG loaded Liposomal Spherical Nucleic Acid for Enhanced Cancer Treatment**

Bo Deng^1^, Bing Ma^1^, Yingying Ma^1^, Pei Cao^2^, Xigang Leng^1^, Pengyu Huang^1^, Tianjiao Ji^2^, Yuanyuan Zhao^3^, Xueguang Lu^3*^, Lanxia Liu^1*^

1. Tianjin Key Laboratory of Biomedical Materials, Key Laboratory of Biomaterials and Nanotechnology for Cancer Immunotherapy, Institute of Biomedical Engineering, Chinese Academy of Medical Sciences & Peking Union Medical College, Tianjin 300192, China

2. CAS Key Laboratory for Biomedical Effects of Nanomaterials & Nanosafety, CAS Center for Excellence in Nanoscience, National Center for Nanoscience and Technology, Beijing 100190, China

3. Key Laboratory of Colloid, Interface and Chemical Thermodynamics, Institute of Chemistry, Chinese Academy of Science, Beijing100190, PR China.

*Corresponding author

Xueguang Lu, Ph.D.

Institute of Chemistry, Chinese Academy of Sciences, No. 2, 1st North Street, Zhongguancun, Beijing100190, PR China.

Email: xueguang@iccas.ac.cn

Lanxia Liu, Ph.D.

Institute of Biomedical Engineering, Peking Union Medical College & Chinese Academy of Medical Sciences, Tianjin 3000192, China

Phone & Fax: +86 (22) 87891191

E-mail: liulanxiabme@163.com


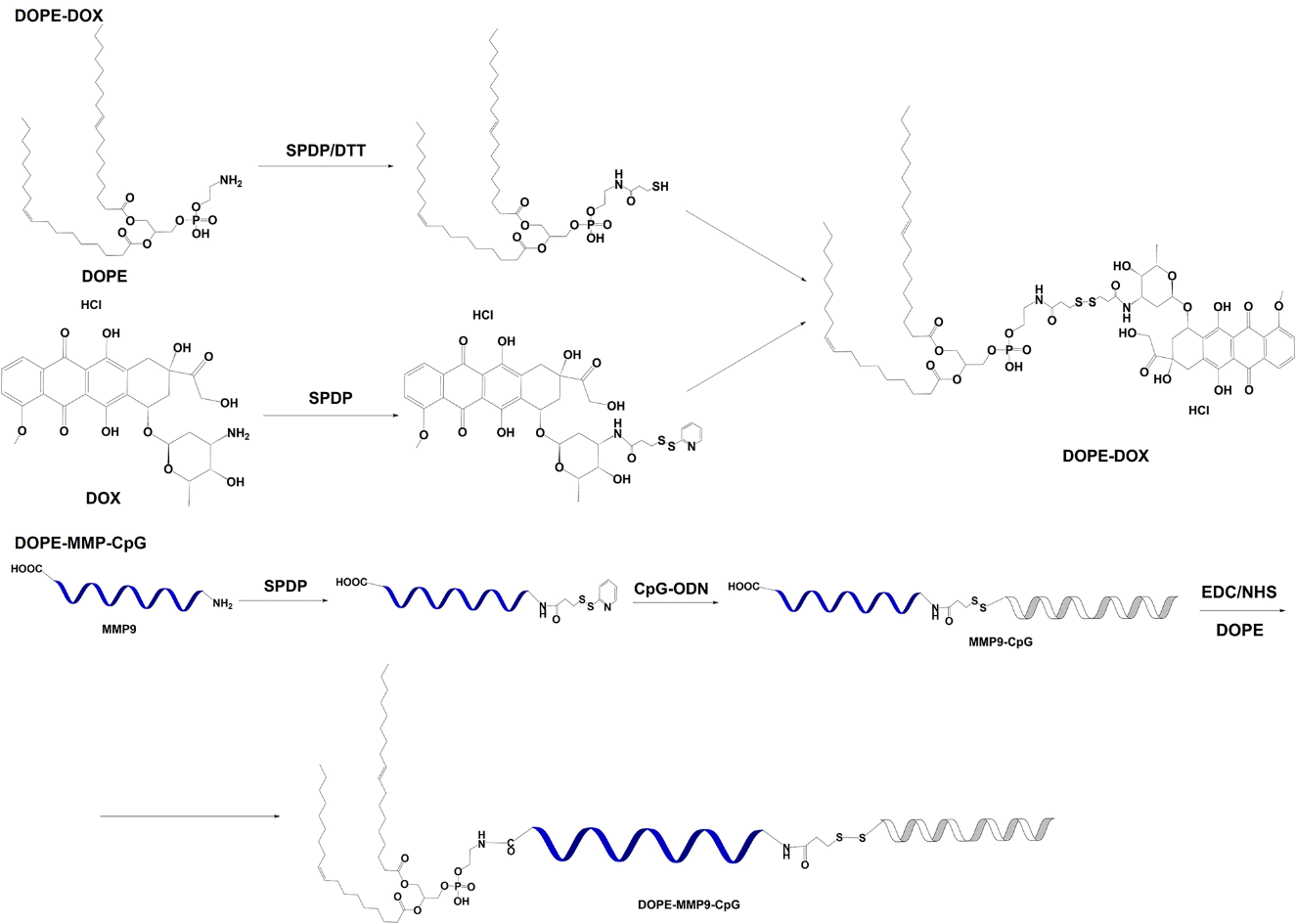


**Fig S1.** Synthetic scheme of DOPE-DOX and DOPE-MMP-CpG.


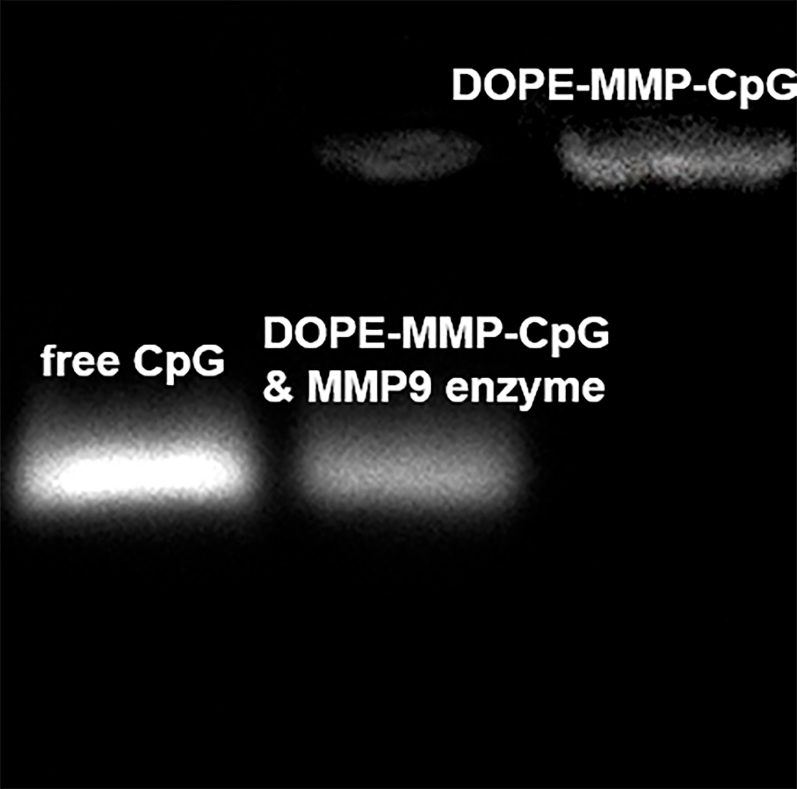


**Fig S2.** The agarose gel electrophoresis image of free CpG, DOPE-MMP-CpG& MMP9 enzyme, and DOPE-MMP-CpG.


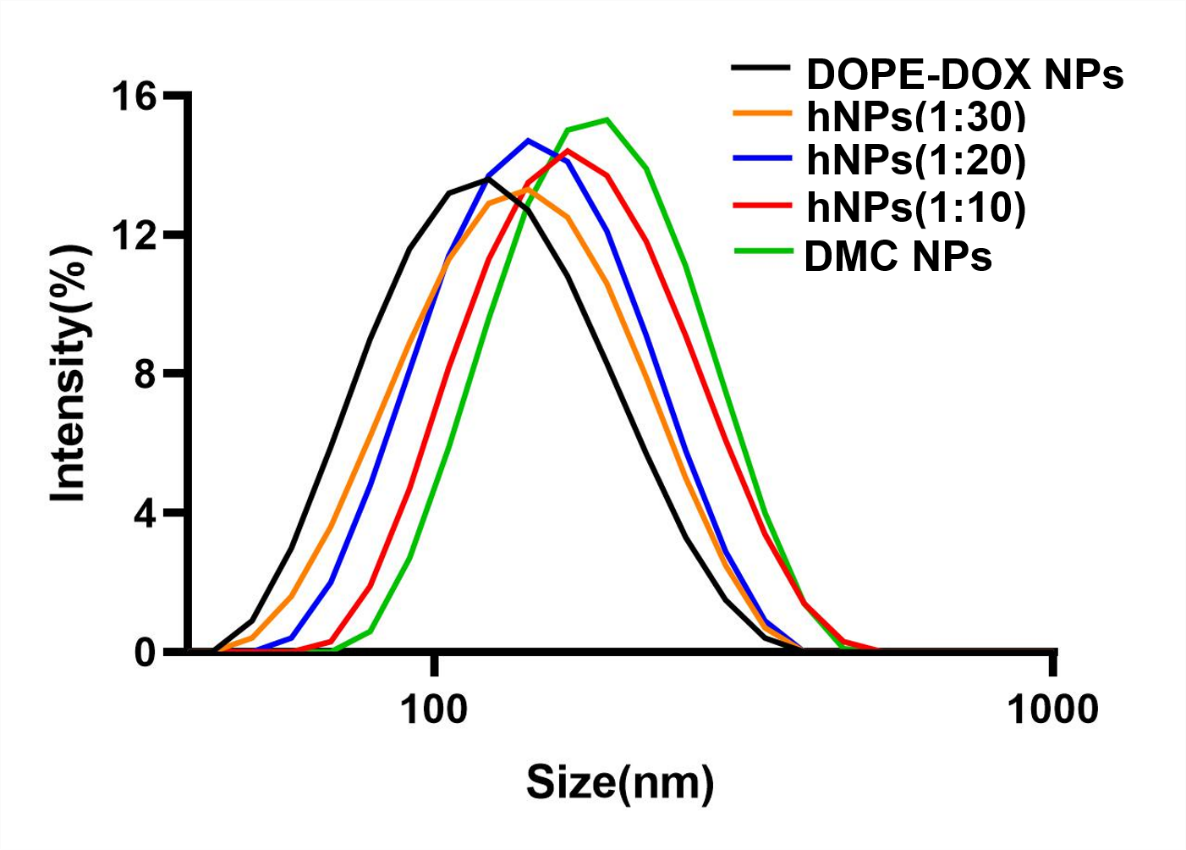


**Fig S3.** DLS measurements of hNPs at different ratios of DOPE-DOX to DOPE-MMP-CpG in water.


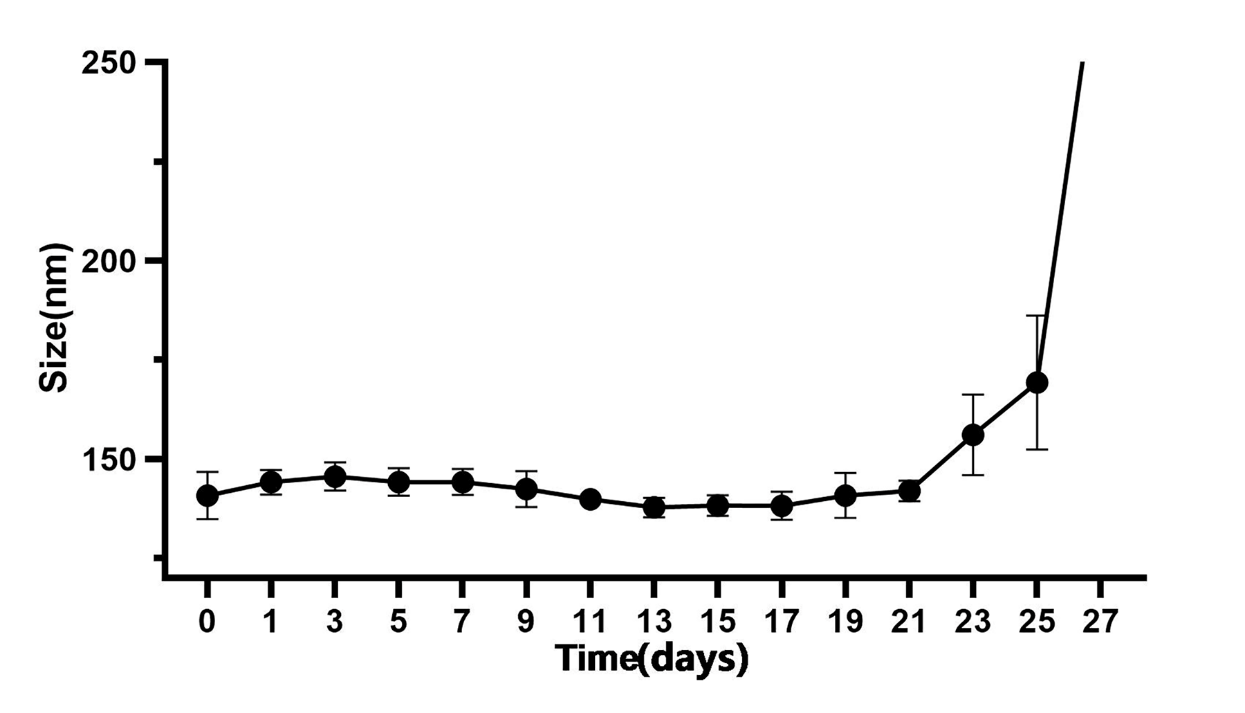


**Fig S4.** DLS measurements of hNPs in PBS at 37 °C. Data represent mean ± SD (n = 3).


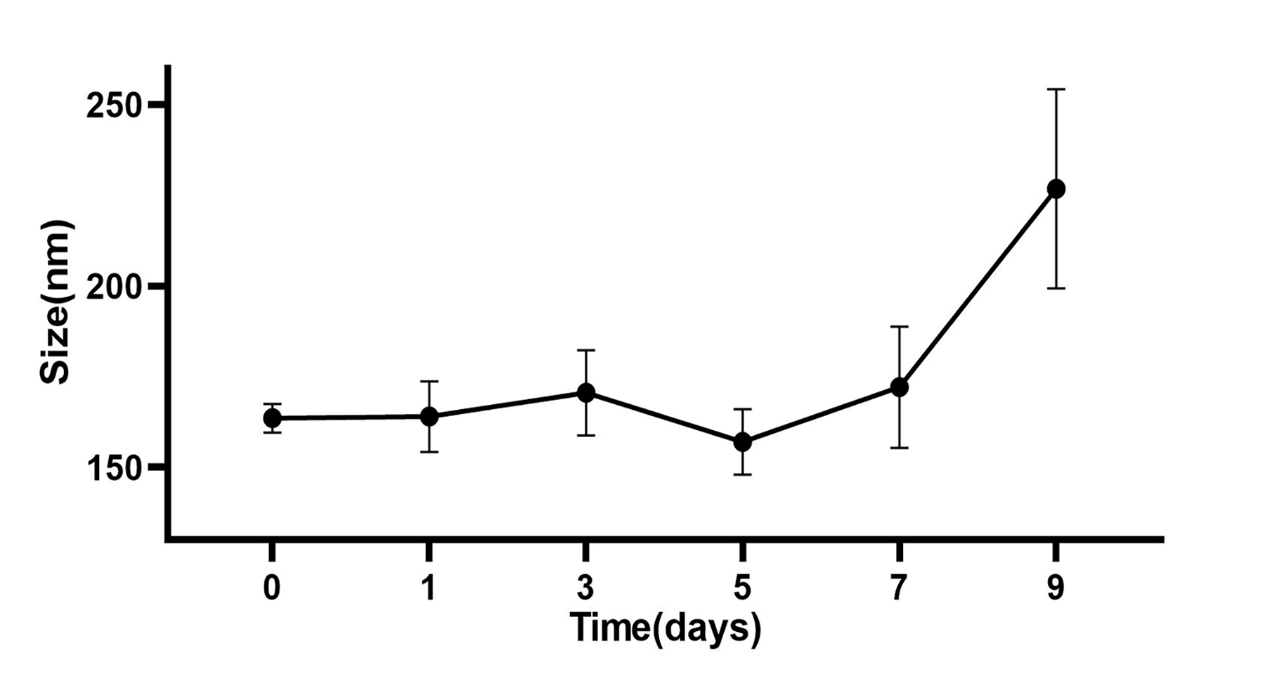


**Fig S5.** DLS measurements of hNPs in RPMI medium containing 10% FBS at 37 °C. Data represent mean ± SD (n = 3).


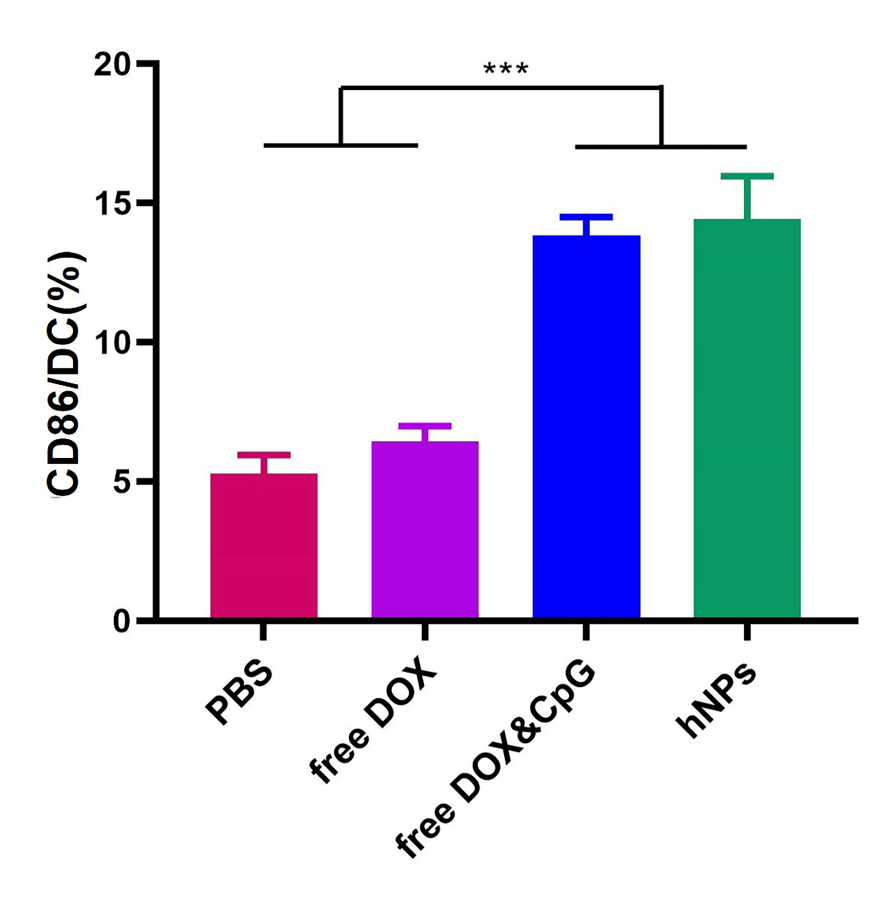


**Fig S6.** BMDCs were incubated with dying tumor cells or debris treated with various formulations for 48h. The expression level of CD86 on BMDC was analyzed by flow cytometry. Data represent mean ± SD (n = 6; ***P < 0.001).


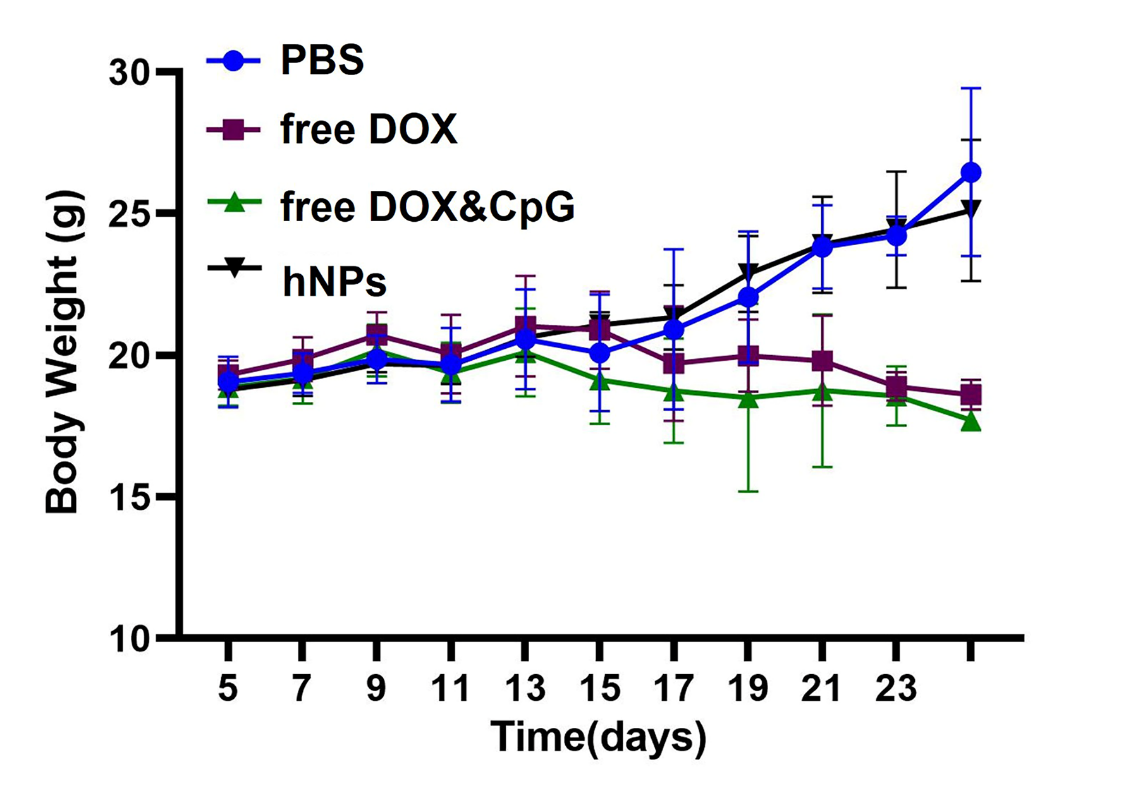


**Fig S7.** Body weight change of mice after treatment with PBS, free DOX, free DOX & CpG, and hNPs, respectively. Data represent mean ± SD (n = 5).
